# Transcriptomic Analysis Reveals Candidate Genes for Female Sterility in Pomegranate Flowers

**DOI:** 10.3389/fpls.2017.01430

**Published:** 2017-08-23

**Authors:** Lina Chen, Jie Zhang, Haoxian Li, Juan Niu, Hui Xue, Beibei Liu, Qi Wang, Xiang Luo, Fuhong Zhang, Diguang Zhao, Shangyin Cao

**Affiliations:** Zhengzhou Fruit Research Institute, Chinese Academy of Agricultural Sciences Zhengzhou, China

**Keywords:** bisexual flowers, functional male flowers, female sterility, *Punica granatum* L., transcriptomic analysis

## Abstract

Pomegranate has two types of flowers on the same plant: functional male flowers (FMF) and bisexual flowers (BF). BF are female-fertile flowers that can set fruits. FMF are female-sterile flowers that fail to set fruit and that eventually drop. The putative cause of pomegranate FMF female sterility is abnormal ovule development. However, the key stage at which the FMF pomegranate ovules become abnormal and the mechanism of regulation of pomegranate female sterility remain unknown. Here, we studied ovule development in FMF and BF, using scanning electron microscopy to explore the key stage at which ovule development was terminated and then analyzed genes differentially expressed (differentially expressed genes – DEGs) between FMF and BF to investigate the mechanism responsible for pomegranate female sterility. Ovule development in FMF ceased following the formation of the inner integument primordium. The key stage for the termination of FMF ovule development was when the bud vertical diameter was 5.0–13.0 mm. Candidate genes influencing ovule development may be crucial factors in pomegranate female sterility. *INNER OUTER* (*INO/YABBY4*) (*Gglean016270*) and *AINTEGUMENTA (ANT)* homolog genes (*Gglean003340* and *Gglean011480*), which regulate the development of the integument, showed down-regulation in FMF at the key stage of ovule development cessation (ATNSII). Their upstream regulator genes, such as *AGAMOUS-like (AG-like)* (*Gglean028014, Gglean026618*, and *Gglean028632*) and *SPOROCYTELESS (SPL)* homolog genes (*Gglean005812*), also showed differential expression pattern between BF and FMF at this key stage. The differential expression of the ethylene response signal genes, *ETR* (*ethylene-resistant*) (*Gglean022853*) and *ERF1/2* (*ethylene-responsive factor*) (*Gglean022880*), between FMF and BF indicated that ethylene signaling may also be an important factor in the development of pomegranate female sterility. The increase in BF observed after spraying with ethephon supported this interpretation. Results from qRT-PCR confirmed the findings of the transcriptomic analysis.

## Introduction

Female sterility is a widespread phenomenon in various plants, such as *Arabidopsis* (Robinson-Beers et al., 1992), tomato (Honma and Phatak, 1964), rice (Li et al., 2006), Japanese apricot (Shi et al., 2011), *Xanthoceras sorbifolia* (Gao et al., 2002), and *Prunus armeniaca* L. (Lillecrapp et al., 1999). There are three main types of female sterility: flowers with no pistil, or only an incomplete pistil; flowers with pistil but lacking normal ovules; and flowers with normal ovules but in which there is abnormal embryo growth following pollination (Li et al., 2006). For fruit crops, female sterility is a double-edged sword. On the one hand, it is inversely proportional to the rate of fruit set, and it therefore seriously reduces fruit yield. On the other hand, it can help assist a plant to allocate limited resources to male and female reproductive function (Bertin, 1982).

Pomegranate (*Punica granatum* L.) is a shrub that is native to central Asia (Holland et al., 2009), it is valued for its juicy aril sacs, which are claimed to be of benefit to human health (Lansky and Newman, 2007; Basu and Penugonda, 2008; Wang et al., 2012; Jaime et al., 2013). Two types of flowers are produced on an individual pomegranate tree: FMF and BF (Holland et al., 2009; Wetzstein et al., 2011). FMFs, which are referred as “female-sterile,” or “bell-shaped,” fail to set fruit and eventually fall from the tree. BFs, on the other hand, posses well-formed pistils and can set fruit; thus they are known as “female-fertile,” “vase-shaped” (Holland et al., 2009; Wetzstein et al., 2011). The proportion of BF correlates with fruit yield, whereas FMF assist in gene dispersal on account of their more efficient pollen production (Tanurdzic and Banks, 2004; Wetzstein et al., 2011). FMF female sterility is caused mainly by abnormal ovule development (Wetzstein et al., 2011). This is also the cause of female sterility in *X. sorbifolia* (Gao et al., 2002) and *Prunus armeniaca* L (Lillecrapp et al., 1999). However, the molecular mechanism of female sterility in fruit crops remains unknown.

In some plant species, such as *Arabidopsis*, cotton, and rice, significant progress in elucidating the molecular mechanism of ovule development has been achieved (Baker et al., 1997; Lee et al., 2006; Kubo et al., 2013). The ABCDE model of flower determination and development has indicated that a specific class of MADS-box genes are key regulators of ovule development (Colombo et al., 1995; Theißen and Saedler, 2001; Tsai and Chen, 2006). The investigation of *agamous* (*AG*) mutant in *Arabidopsis* showed a complete lack of both stamens and carpels, which indicate the importance of *AG* in specifying carpel identity (Bowman et al., 1991). *AG* is a key regulator of carpel development, through controlling the expression of other genes with regulatory functions (Ó’Maoiléidigh et al., 2013). In particular, *AG* subfamily genes positively regulate the expression of *SPL* (Ito et al., 2004). *SPL* regulates ovule development by repressing the expression of a number of genes that are important in ovule development, such as *ANT, BEL1* and *INO*, but the mechanism of *SPL* repression of these genes remains unknown (Sieber et al., 2004; Wei et al., 2015). *ANT* and *INO* are key regulators of ovule integument formation (Grossniklaus and Schneitz, 1998). *ANT* is a member of (APETALA2)/EREBP (Ethylene Responsive Element Binding Protein) multi-gene family that regulates the formation of ovule integument primordium by controlling cell growth and number (Mizukami and Fischer, 2000; Shigyo et al., 2006). *ANT* contains two AP2 domains homologous with the DNA binding domain of ethylene response element binding proteins (EREBPs) which was most surely involved in ethylene signal transduction (Klucher et al., 1996; Cucinotta et al., 2014). Previous studies have demonstrated the roles of ethylene in regulating sex determination and increasing the number of female flowers in cucumber and orchid as well as restoring ovule development in the transformed tobacco (Iwahori et al., 1970; De Martinis and Mariani, 1999; Yamasaki et al., 2003; Papadopoulou et al., 2005; Boualem et al., 2008; Tsai et al., 2008). Consistent with *ANT, AP2* also belongs to AP2 subfamily and encodes a transcription factor for putative protein which was same as ethylene responsive factor, ERF protein, that can inhibit the expression of *AG* gene (Pinyopich et al., 2003; Licausi et al., 2013). *AG* family genes are important upstream regulators of ovule development (Dreni and Kater, 2014).

The formation of pomegranate FMF has been associated with abnormal ovule development, but the stage at which ovule abortion occurs, and the causes of abortion, remain unknown. In this study, by means of morphological observations using SEM, we have identified the key developmental stage for ovule abortion. Then, through a combination of RNA sequencing and ethephon treatment, we have identified the candidate genes responsible for female sterility. Our results are important in shedding light on the mechanism of gynoecia development in pomegranate.

## Materials and Methods

### Plant Growth and Sample Collection

Pomegranate flowers were collected from 10-year-old “Tunisiruanzi” trees grown in nursery of the Zhengzhou Fruit Research Institute located in Zhengzhou, Henan, China, and managed using conventional methods. BF and FMF were separated based on the morphology of the ovary, which in FMF is atrophied (Holland et al., 2009). The ovules of FMF cease differentiation when their BVD was 5.0–13.0 mm. A total of 18 accessions were sequenced, comprising three BF and three FMF, and including three biological replicates. To describe the accessions clearly, we used TNSI, TNSII, and TNSIII represent BF buds when their BVD was 3.0–5.0, 5.1–13.0, 13.1–25.0 mm, respectively. Similarly, ATNSI, ATNSII, and ATNSIII represented the FMF buds when their BVD was 3.0–5.0, 5.1–13.0, 13.1–25.0 mm, respectively. Following collection, all samples were immediately frozen in liquid nitrogen and stored at -80°C.

### RNA Extraction and Sequencing

Total RNA extraction was performed using the CTAB (Cetyltrimethyl Ammonium Bromide) method. RNA purity was checked using a NanoDrop^®^ 2000 instrument (Thermo, Wilmington, CA, United States). RNA concentration and integrity were measured using the RNA Nano 6000 Assay Kit with the Agilent Bioanalyzer 2100 system (Agilent Technologies, Santa Clara, CA, United States). Library preparation and sequencing reactions were carried out by 1Gene, Corp. (Hangzhou, China)^1^ according to the relevant manufacturer’s instructions (Illumina, San Diego, CA, United States).

### Library Preparation for Transcriptome Sequencing

A total amount of 5 μg RNA per sample was used as input material for the RNA sample preparations. Sequencing libraries were generated using a NEBNext^®^ Ultra^TM^ RNA Library Prep Kit for Illumina^®^ (NEB, Ipswich, MA, United States) following the manufacturer’s recommendations, and index codes were added to attribute sequences to each sample. Briefly, mRNA was purified from total RNA using poly-T oligo-attached magnetic beads. Fragmentation was carried out using divalent cations under elevated temperature in NEB-Next^®^ First Strand Synthesis Reaction Buffer (5×) (NEB, Ipswich, MA, United States). First-strand cDNA was synthesized using random hexamer primer and M-MuLV reverse transcriptase (RNase H-). Second-strand cDNA synthesis was subsequently performed using DNA polymerase I and RNase H. Remaining overhangs were converted into blunt ends using exonuclease/polymerase activities. Following adenylation of the 3′-ends of the DNA fragments, a NEBNext adaptor with a hairpin loop structure was ligated to prepare the fragments for hybridization. In order to select cDNA fragments of preferentially 250–300 bp in length, the library fragments were purified using an AMPure XP system (Beckman Coulter, Beverly, MA, United States). The size-selected, adaptor-ligated cDNA was incubated with 3 μl of USER Enzyme (NEB) at 37°C for 15 min. PCR was then performed with Q5 Hot Start HiFi DNA polymerase (NEB, Ipswich, MA, United States), Universal PCR primers and Index (X) Primer. Finally, the PCR products were purified (AMPure XP system) and library quality was assessed using the Agilent Bioanalyzer 2100 system.

### Clustering and Sequencing

Clustering of the index-coded samples was performed on a cBot Cluster Generation System using a TruSeq PE Cluster Kit v4-cBot-HS (Illumina) according to the manufacturer’s instructions. After cluster generation, the library preparations were sequenced on an Illumina Hiseq 2500 platform and paired-end reads were generated.

### Data Analysis

#### Quality Control

Raw data (raw reads) in FASTQ format were firstly processed through in-house Perl scripts. In this step, clean data (clean reads) were obtained by removing reads containing adapter, reads containing poly-N and low-quality reads from raw data. At the same time, the Q20, Q30 and GC content of the cleaned data were calculated. All the downstream analyses were based on the high-quality cleaned data.

#### Mapping Reads to the Reference Genome

Reference genome and gene model annotation files were obtained form our lab (unpublished data). The index of the reference genome was built using Bowtie v2.2.3 and paired-end clean reads were aligned to the reference genome using TopHat v2.0.12 (Langmead and Salzberg, 2012; Kim et al., 2013). We selected TopHat as the mapping tool because TopHat can generate a database of splice junctions based on the gene model annotation file and thus produces a better mapping result than other non-splice mapping tools.

#### Quantification of Gene Expression Level

Reads per kilobase of transcript per million reads mapped (RPKM) of each gene was calculated based on the length of the gene and the reads count mapped to this gene. RPKM, takes account simultaneously of the effect of sequencing depth and gene length for the reads count, and is currently the most commonly used method of estimating gene expression levels (Trapnell et al., 2010).

#### Differential Expression Analysis

Prior to differential gene expression analysis, for each sequenced library, the read counts were adjusted using the edgeR program package with a one-scale normalizing factor. Differential expression analysis of the two groups (three biological replicates per group) was performed using the DESeq R package (1.20.0)^2^. DESeq provides statistical routines for determining differential expression in digital gene expression data, using a model based on the negative binomial distribution. The resulting *P*-values were adjusted using the Benjamin and Hochberg approach for controlling the false discovery rate (Benjamini and Hochberg, 1995). Genes with an adjusted *P*-value < 0.05 found by DESeq were assigned as differentially expressed. An adjusted *P*-value of 0.005 and a log2 (fold change) of 1 were set as the threshold for significantly differential expression.

#### Go and KEGG Enrichment Analysis of Differentially Expressed Genes

Gene Ontology enrichment analysis of DEGs was carried out using topGO, in which gene length bias was adjusted. GO terms with a adjusted *P*-value < 0.05 were considered to be significantly enriched in DEGs.

The Kyoto Encyclopedia of Genes and Genomes (KEGG) is a database resource for understanding the high-level functionalities of a biological system – such as the cell, the organism, and the ecosystem – from molecular-level information, especially data from large-scale molecular datasets generated by genome sequencing and other high-throughput experimental technologies^3^. R software was used to test the statistical enrichment of DEGs in KEGG pathways.

#### Gene Co-expression Network Analysis

Gene co-expression network analysis was performed using the weighted gene correlation network analysis (WGCNA) (v1.29) package in R (Langfelder and Horvath, 2008). Only genes with a RPKM value greater than 2 in all samples were analyzed. The modules were obtained using the automatic network construction function “blockwiseModules” with default settings, TOMType “signed,” minModuleSize = 30, and mergeCutHeight = 0.25. The total connectivity and intramodular connectivity (function “softConnectivity”), kME, and kME-*p*-value were calculated. The correlations between modules and four traits (BVD, SL, OTD, and OVD) were calculated by the “cor” function in R, with significance calculated using corPvalueStudent. The correlation between modules and samples was undertaken using module Eigengenes in WGCNA. The networks were visualized using Cytoscape _v.3.0.0^4^.

### Real-time RT-PCR Analysis

To verify the RNA-Seq analysis, we performed qRT-PCR analysis of the expression of nine genes, which included two genes (*ETR, ERF1/2*) related to the ethylene response and seven genes (three *AG*, two *SPL*, one *ANT* and one *INO*) related to pistil development. The *actin* gene was used as a reference (**Supplementary Table S9**). RNA was extracted as described above and cDNA was synthesized using a TIANscript RT Kit (TIANGEN, Beijing, China). PCR was performed using 2 × SYBR Green Real-time PCR Master Mix (Roche) on a real-time PCR instrument (Roche 480, Basle, Switzerland), with the following program: pre-incubation at 95°C for 5 min, then followed by 45 cycles of 95°C for 10 s, 60°C for 10 s, and 72°C for 10 s. The ddCt method was used to calculate gene expression levels (Livak and Schmittgen, 2001). Primer sequences are listed in **Supplementary Table S9**.

### Scanning Electron Microscopy (SEM)

Pistil of FMF and BF were collected when their BVD were 3.0–5.0, 5.1–10.0, 10.1–13.0, 13.1–15, 15.1–18.0, 18.1–20.0, 21.1–25.0, and 25.1–35.0 mm (blooming). Samples were fixed in 2.5% glutaraldehyde (pH = 7.4) for > 1 week at 4°C. Following fixation, they were dehydrated using an ethanol series [30% ethanol, 20 min; 50% ethanol, 20 min; 70% ethanol, 20 min; 100% ethanol, 30 min (twice)]. The dehydrated samples were then dried in a critical-point drying apparatus (Quorum, England). Dried samples were mounted on stubs and sputter-coated with gold (FEI, America) and observed under a SEM (FEI Quanta 250, America) in Henan University. Ten floral buds were observed in each stage.

### Morphology of Bisexual Flowers (BF) and Functional Male Flowers (FMF)

Flower buds at different stages were collected based on BVDs. Measurements of flower BVD, OTDs, SL were made using a Dsect mirror (DS-Fiec, Nikon) or by vernier caliper. Ten biological replicates were measured for each stage. Difference significance analysis between BF and FMF using independent-samples *t*-test, *P-*value represent significance of difference.

### Treatment with Plant Growth Regulator

“Tunisiruanzi” pomegranate trees were sprayed with an aqueous solution of ethephon at concentrations of 150, 200, and 250 mg/L, with three biological replications per treatment. Control sprayings were with water alone. Each tree was sprayed with 1. Five liter aqueous solution of ethephon or water (control). Treatments were sprayed on whole trees once, including both floral buds and leaves at a BVD of 3.0–5.0 mm (April 22, 2016), at which stage it is not yet possible to distinguish BF from FMF.

## Results

### Morphology of Pomegranate Bisexual Flowers (BF) and Functional Male Flowers (FMF)

**FIGURE 1:**
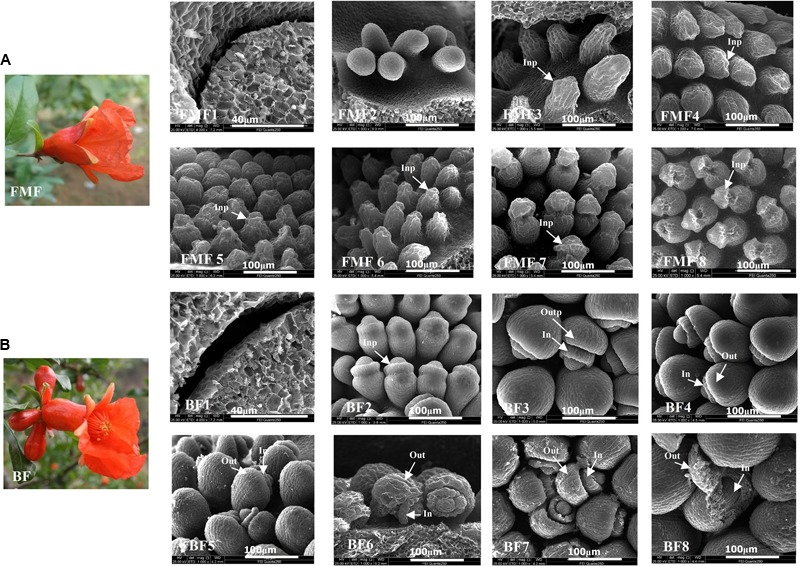
Scanning electron micrographs (SEM) and photographs showing the developmental stages of the functional male flowers (FMF) ovule and bisexual flowers (BF) ovule. **(A)** The morphologies of FMF, **(B)** the morphologies of BF. **(FMF1–8)** The ovule development of FMF when their bud vertical diameter (BVD) was 3.0–5.0, 5.1–10.0, 10.1–13.0, 13.1–15.0, 15.1–18.0, 18.1–21.0, and 21.1–25.0 mm, flowering time (BVD ≥ 25.1 mm). **(BF1–8)** represented the ovule development of BF when their BVD was 3.0–5.0, 5.1–10.0, 10.1–13.0, 13.1–15.0, 15.1–18.0, 18.1–21.0, and 21.1–25.0 mm, flowering time (BVD > 25.1 mm).

The morphology of FMF is bell-shaped, whereas that of BF is vase-shaped (**Figures 1 FMF, 1BF**). Pistils of FMF and BF were collected when their BVDs were: 3.0–5.0 mm (**Figures 1FMF1, 1BF1**); 5.1–10.0 mm (**Figures 1FMF2, 1BF2**); 10.1–13.0 mm (**Figures 1FMF3, 1BF3**); 13.1–15.0 mm (**Figures 1FMF4, 1BF4**); 15.1–18.0 mm (**Figures 1FMF5, 1BF5**); 18.1–20.0 mm (**Figures 1FMF6, 1BF6**); 20.1–25.0 mm (**Figures 1FMF7, 1BF7**) and during flowering (BVD ≥ 25.1 mm) (**Figures 1FMF8, 1BF8**). The dynamics of ovule development of FMF and BF were observed (**Figure 1**).

There were no obvious differences observed between BF1 and FMF1 (BVD 3.0–5.0 mm), at this stage, the placenta had formed but no ovule primordia were observable. Stage BF2 (BVD 5.1–10.0 mm) was characterized by the formation of inner integument primordia. However, in FMF2, though the ovules were enlarged, no inner integument primordia had formed. At BF3 (BVD 10.1–13.0 mm), outer integument primordia formed, the inner integument showed an increase in the number of cell layers and became symmetrically enlarged, growing parallel to the nucellus by anticlinal division and cell elongation (Reiser and Fischer, 1993). By contrast, at FMF3 (BVD 10.1–13.0 mm), inner integument primordium formed in part of the ovules, but no inner integument extension and outer integument primordia were observed, ovules displayed wilting. Thereafter, ovules undergo wilting during stage FMF4–8. In BF, on the other hand, the integument continued to enlarge during stage BF4 (BVD 13.1–15.0 mm); and during the subsequent stages BF5–7 (BVD 15.1–25.0 mm), the outer integument exhibited a gradient of cell division, which showed maximal growth on the abaxial side and almost no growth on the adaxial side (Robinson-Beers et al., 1992). By the reason of this asymmetric growth, by stage BF8 (when the flowers were in bloom), the outer integument completely enclosed the inner integument and the nucleus. From this comparison of FMF and BF, it was apparent that early ovule development in FMF was indistinguishable from that in BF. One of the reasons for pistil abortion in FMF may be the termination of ovule development after stage FMF3 (BVD 10.1–13.0 mm).

**FIGURE 2:**
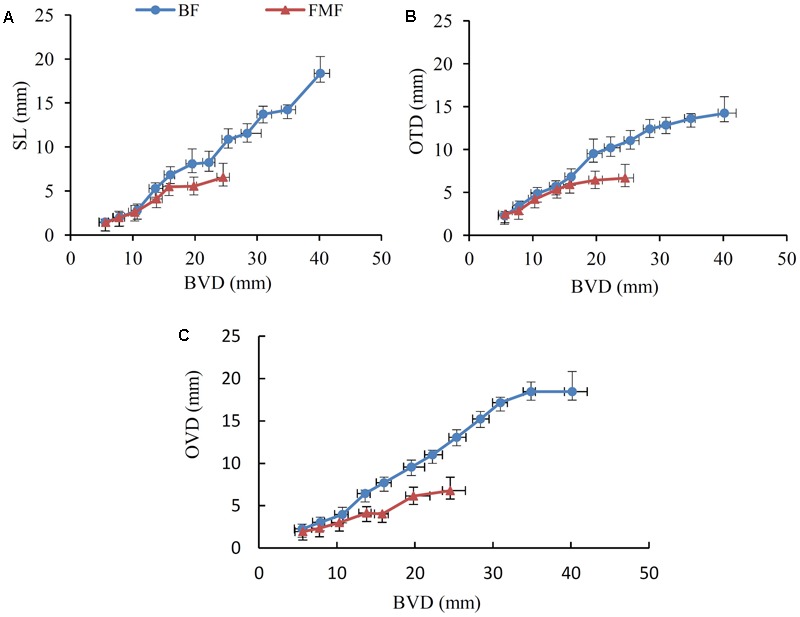
Changing curve of style length (SL), ovary transverse diameter (OTD), and ovary vertical diameter (OVD) of BF and functionally male flowers of ‘Tunisiruanzi’ with BVD. **(A)** Changing curve of SL with BVD. **(B)** Changing curve of OTD with BVD. **(C)** Changing curve of OVD with BVD. SL, style length; OTD, ovary transverse diameter; OVD, ovary vertical diameter. Transverse error bars means standard deviation of BVD, vertical error bars means standard deviation of SL OVD and OTD. Each data point represents the mean value of 10 technical replicates.

At flowering time, FMF were smaller than BF (**Figure 1A**). FMF bloomed at a BVD of 24.54 ± 1.59 mm (**Figures 2A–C**), a SL of 7.97 ± 1.0 mm (**Figure 2A**), an OTD of 6.67 ± 1.33 mm (**Figure 2B**), and an OVD of 14.22 ± 1.85 mm (**Figure 2C**). In contrast, BF bloomed at a BVD of 40.18 ± 2.37 mm (**Figures 2A–C**), a SL of 18.34 ± 1.48 mm (**Figure 2A**), an OTD of 14.22 ± 1.85 mm (**Figure 2B**), and an OVD of 14.22 ± 1.85 mm (**Figure 2C**). Thus, during the flowering period, the OTD (*P* < 0.01), the SL (*P* < 0.05) and the OVD (*P* < 0.01) were clearly shorter in FMF than in BF (**Supplementary Table S1**).

Remarkably, in FMF, the increase in OTD, SL and OVD began to decline when BVD grow into 10.32 ± 0.77–15.82 ± 0.13 mm (**Figures 2A,B**), suggesting OTD, SL and OVD can be indicators for female sterility. The SEM results showed that the key stage at which ovule abortion occurred when BVD was 10.0–13.0 mm (**Figure 1**). The data further prove the key stage of pomegranate female sterility was 10.0–13.0 mm in BVD.

### Transcriptome Sequencing of Pistils

**FIGURE 3:**
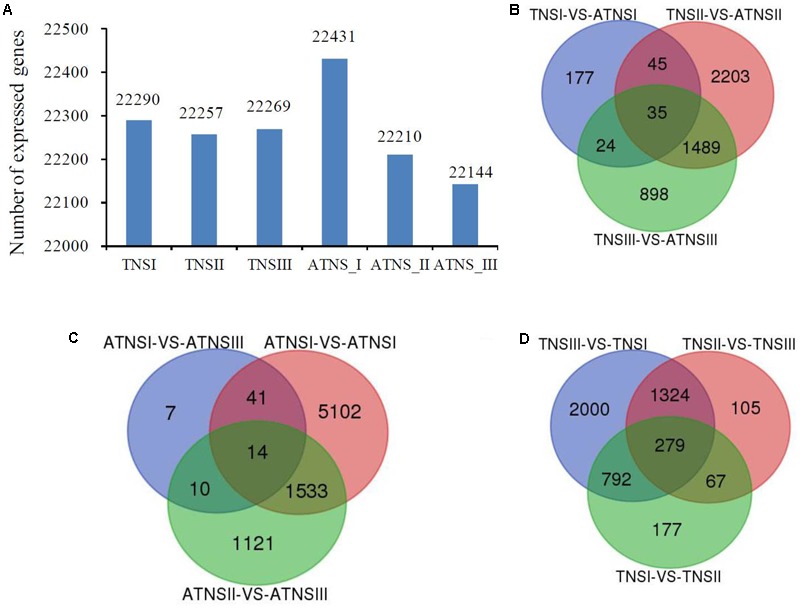
The number of different expressed genes. **(A)** Different number of expressed genes in different stages. **(B–D)** Unique and shared DEGs among different stages for both BF and FMF. TNSI, TNSII, TNSIII represent pistil of BFs when their BVD was 3.0–5.0, 5.1–13.0, and 13.1–25.0 mm, respectively. ATNSI, ATNSII, ATNSIII stand for pistil of FMFs when their BVD was 3.0–5.0, 5.1–13.0, and 13.1–25.0 mm, respectively. **(B)** The number of DEGs between ATNSI -VS- TNSI, ATNSII -VS- TNSII, ATNSIII -VS- TNSIII. **(C)** The number of DEGs between ATNSI -VS- ATNSII, ATNSI -VS- ATNSIII, ATNSII -VS- ATNSIII; **(D)** The number of DEGs between TNSI -VS- TNSII, TNSI -VS- TNSIII, TNSII -VS- TNSIII; Venn diagrams were drawn using a online tool Venn Diagrams (http://bioinformatics.psb.ugent.be/webtools/Venn/). BF, bisexual flowers; FMF, functional male flowers. VS in **B–D** represent versus.

In order to denote the accessions clearly, we used TNSI, TNSII, and TNSIII represent the BF buds’ pistils when their BVD was 3.0–5.0, 5.1–13.0, 13.1–25.0 mm, respectively. Similarly, the designations ATNSI, ATNSII, and ATNSIII were used to represent the FMF buds’ pistils when their BVD was 3.0–5.0, 5.1–13.0, 13.1–25.0 mm, respectively. There were three replicates for each developmental stage. In total, 18 accessions were used for transcriptomic sequencing. Sequencing of 18 accessions by Illumina platform obtained a total of 841 million clean reads of sequence, with an average of 46.7 million clean reads and 92.08% of mapped rate for each accession (**Supplementary Table S2**). The raw data were uploaded to NCBI Sequence Read Archive (SRA), a total of 18 samples with accession numbers SRX2735567-SRX2735584 were uploaded^5^. Pearson r^2^ correlation values for all replications were used to evaluate the consistency of the raw data. Most of them varied from 0.85 to 1.0, and data with a value <0.80 were removed prior to subsequent analysis (Supplementary Figure S1). The data showed that the sequencing quality was high enough for further analysis (Supplementary Figure S1). A total of 25,959 genes were detected, with only a small variation between accessions (**Supplementary Table S2**). The highest number of expressed genes was 22,431, obtained from ATNSI (**Figure 3A**), while the lowest number (22,144) was from ATNSIII (**Figure 3A** and **Supplementary Table S2**).

### Differentially Expressed Genes and Enrichment Analysis

A total of 9662 unique DEGs were obtained. Between TNSI and ATNSI, the number of DEGs was 281, whereas it was 3,772 between TNSII and ATNSII. However, the number decreased to 2,446 when comparing TNSIII with ATNSIII (**Figure 3B**). Moreover, a total of 7,828 DEGs were detected between the FMF different development stages (ATNSI-ATNSII, ATNSI-ATNSIII, and ATNSII-ATNSIII) and 4,744 DEGs were detected between three stages of BF development (TNSI-TNSII, TNSI-TNSIII, and TNSII-TNSIII) (**Figures 3C,D**). The DEGs were listed in **Supplementary Table S3**.

An enrichment analysis was performed to determine whether the genes (DEGs) that were differentially expressed between FMF and BF were markedly associated with specific pathways or biological processes. The DEGs were characterized using the GO and KEGG databases. The GO terms comprise three categories: molecular function, cellular component, and biological process. A total of 4,781 unique genes were enriched between FMF and BF (TNSI-ATNSI, TNSII-ATNSII, and TNSIII-ATNSIII). For the TNSI-ATNSI DEGs, the classification terms that showed enrichment included “response to hormone” (GO:0009725), “cell death” (GO:0008219), “signal transduction” (GO:0007165), “regulation of programmed cell death” (GO:0043067), “response to ethylene” (GO:0009723), “ethylene biosynthetic process” (GO:0009693), and “regulation of cellular process (GO:0050794)” (**Supplementary Table S3**). In contrast, for the TNSII-ATNSII DEGs, the enriched classification terms were “cell proliferation” (GO:0008283), “DNA methylation” (GO:0006306, GO:0044728), “regulation of cell cycle” (GO:0051726), “cell cycle” (GO:0007049), “mitotic cell cycle process” (GO:1903047), “G2/M transition of mitotic cell cycle” (GO:0000086), “cell division” (GO:0051301), “regulation of gene expression, epigenetic” (GO:0040029), and “nuclear division” (GO:0000280) (**Supplementary Table S3**). For the TNSIII-ATNSIII comparison, the enriched terms belonged to categories including “cell cycle” (GO:0007049), “cell division” (GO:0051301), “mitotic cell cycle process” (GO:1903047), “regulation of growth” (GO:0040008), “flower development” (GO:0009908), “nuclear division” (GO:0000280), “reproductive structure development” (GO:0048608), and “floral organ morphogenesis” (GO:0048444) (**Supplementary Table S3**). From the SEM results, ovule development of FMF ceased after the formation of the inner-integument primordium. Thus, the DEGs in the enriched categories such as “cell cycle,” “cell division,” “floral development,” and “floral organ morphogenesis” may be candidate genes associated with pistil abortion. The enrichment analysis revealed a total of 114 DEGs related to flower development (**Supplementary Table S3**).

Spraying with ethephon can increase the proportion of BF (Chaudhari and Desai, 1993). Consistent with this, the category “response to ethylene” (GO:0009723) was enriched between BF and FMF, consistent with the role of ethylene in pomegranate female sterility.

Pathway assignment was carried out by KEGG to analyze the biological functions of DEGs. A total of 1,302 unique DEGs between FMF and BF were mapped into KEGG pathways, containing 41, 809 and 452 DEGs between TNSI-ATNSI, TNSII-ATNSII and TNSIII-ATNSIII comparison, respectively. For the TNSI-ATNSI comparison, pathways related to “plant hormone signal transduction” (ko04075), “plant–pathogen interaction” (ko04626), and “biosynthesis of secondary metabolites” (ko01110) were enriched (**Supplementary Table S4**). For the TNSII-ATNSII comparison, the main pathways included “biosynthesis of secondary metabolites” (ko01110), “plant hormone signal transduction” (ko04075), and “metabolic pathways” (ko01100) (**Supplementary Table S4**). Similarly, “plant hormone signal transduction” (ko04075), “metabolic pathways” and “biosynthesis of secondary metabolites” (ko01110) were enriched in the TNSIII-ATNSIII comparison (**Supplementary Table S4**). The process of plant hormone signal transduction (Supplementary Figure S3) was concluded to be a putative pathway affecting pomegranate FMFs’ ovule development.

### Gene Co-expression Network Analysis Using WGCNA

**FIGURE 4:**
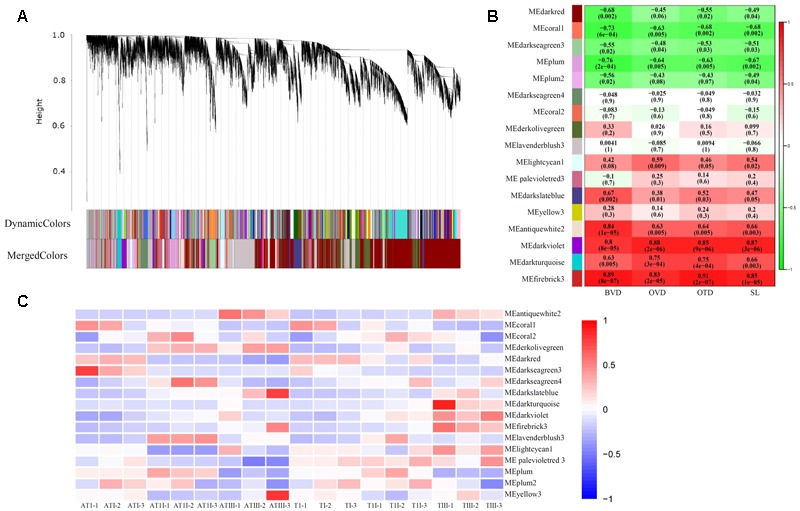
Genes co-expression network analysis with WGCNA. **(A)** Hierarchical cluster tree of different co-expression modules. Each branch constitute one module, and each leaf in the branches represented one gene. A total of 17 distinct modules were labeled by different colors. **(B)** Module-trait association. Each row corresponds to a module. Each column amount to a trait. BVD, bud vertical diameter; OTD, ovary transverse diameter; OVD, ovary vertical diameter; SL, style length. The color at the right represented the size of correlation coefficient, the digital labels in the box in **B** stand for correlation coefficient (r) (upside) and test value (*P*-value) (bottom). Those modules with *r* > 0.6, *P*-value < 0.05 were selected to discuss. **(C)** Module-tissue association. Each row corresponds to a module. Each column amount to a tissue. The color of each box at the row/column intersection showed the size of correlation coefficient between the module and the tissue type. ATI, ATII, ATIII represented pistil of FMF when their BVD was 3.0–5.0, 5.1–13.0, and 13.1–25.0 mm. TI, TII, TIII represented pistil of BF when their BVD was 3.0–5.0, 5.1–13.0, and 13.1–25.0 mm.

Constructing a dendrogram, a total of 17 distinct modules were obtained, in which each tree branch formed a module, and each leaf in the branch represented one gene (**Figure 4A** and **Supplementary Table S5**). The correlation between different modules were showed in Supplementary Figure S2. Genes in different modules were characterized by GO (**Supplementary Table S6**). From the measurement of the various different indices for FMF and BF, we determined differences in OTD, OVD, and SL as the growth of BVD between FMF and BF (**Figure 2**). The associations between these parameters and the 17 distinct modules were then compiled (**Figure 4B**). The modules related to SL are those named MEantiquewhite2, MEdarkviolet, MEdarkturquoise and MEfirebrick 3 (*r* ≥ 0.6) (**Figure 4B**); the same correlations were seen for OTD and OVD (**Figure 4B**); the modules related to BVD are those named MEdarkslateblue, MEantiquewhite2, MEdarkviolet, MEdarkturquoise and MEfirebrick 3 (*r* ≥ 0.6) (**Figure 4B**). According to the GO enrichment, “single-organism process” (GO:0065007), “organic substance metabolic process” (GO:0071704), “biological regulation” (GO:0065007), “organic substance biosynthetic process” (GO:0044711), “small molecule metabolic process” (GO:0044281), “phosphate-containing compound metabolic process” (GO:0006796), “lipid metabolic process” (GO:0006629), “lipid biosynthetic process” (GO:0008610) and “organonitrogen compound metabolic process” (GO:1901564) were also enriched in these modules (**Supplementary Table S6**). Genes related to flower development were revealed by the association between modules and tissues (**Figure 4C**). Gene modules showing differential expression between FMF and BF are named MElavenderblush 3, MElightcyan1, MEdarkred, MEdarkolivegreen, and MEpalevioletred 3 (**Figure 4C**). These modules were enriched mainly in the categories “cellular process” (GO:0009987), “single-organism process” (GO:0044699), and “metabolic process” (GO:0008152). Processes associated with flower development were also enriched (GO:0048573, GO:0009911, GO:0010227, GO:0048573, GO:0009911, GO:0009908, GO:0009909, GO:0048574) (**Supplementary Table S6**). A total of 20 genes related to flower development were enriched by the module-tissues association (Supplementary Figure S4 and **Table S7**). Analysis of the association between modules and tissues revealed enrichment of 20 genes related to flower development (Supplementary Figure S3 and **Table S7**).

### Genes Related to Ovule Development

**FIGURE 5:**
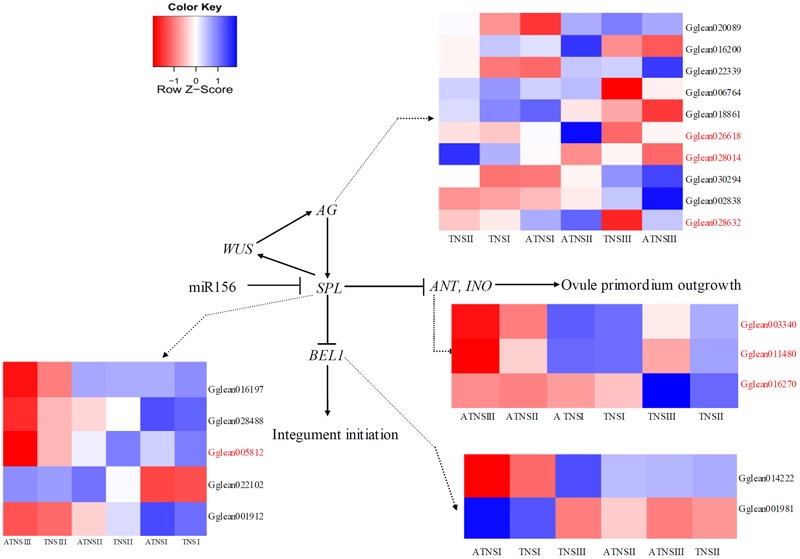
Expression of candidate genes involved in ovule development. Gene expression was measured by log2Ratio. ATNSI, ATNSII, ATNSIII represented pistil of FMF when their BVD was 3.0–5.0, 5.1–13.0, and 13.1–25.0 mm. TNSI, TNSII, TNSIII represented pistil of BF when their BVD was 3.0–5.0, 5.1–13.0, and 13.1–25.0 mm. Gene ID highlighted in red represent DEGs in ATNSII-TNSII comparison. Different colors from blue to red showed the relative log2 (expression ratio).

Abnormal ovule development was the main factor responsible for the abortion of pomegranate flowers. Ovule development comprises several processes: primordia initiation, specification of identity, pattern formation, and eventually morphogenesis and cellular differentiation (Grossniklaus and Schneitz, 1998). The pathway of regulation of *Arabidopsis* ovule development was showed in **Figure 5**, and key regulators include *AGAMOUS* (Yanofsky et al., 1990; Drews et al., 1991), *SPL* (Ito et al., 2004), *INO* (L’eon-Kloosterziel et al., 1994), *ANT* and *BEL1 (*Brown et al., 2010*).* A total of ten *AGAMOUS*-like DEGs (*Gglean016200, Gglean018861, Gglean020089, Gglean026618, Gglean028014, Gglean028632, Gglean030294, Gglean002838, Gglean006764*, and *Gglean022339*), five *SPL* homolog genes (*Gglean016197, Gglean022102, Gglean028488, Gglean001912*, and *Gglean005812*), two *ANT* homolog genes (*Gglean003340* and *Gglean011480*), two *BEL1* homolog genes (*Gglean014222* and *Gglean001981*), and one *INO* homolog gene (*Gglean016270*) involved in ovule development were differential expressed between BF and FMF (**Figure 5** and **Supplementary Table S8**). In the TNSII-ATNSII comparison, which was the key stage for pistil abortion in pomegranate FMF, three *AG*-like genes (*Gglean026618, Gglean028632*, and *Gglean028014*), one *SPL* homolog gene (*Gglean005812*), two *ANT* homolog genes (*Gglean003340* and *Gglean011480*), and one *INO* homolog gene (*Gglean016270*) showed significantly different expression levels (**Supplementary Table S8**). The results indicated that these genes may be the main regulators for the formation of FMF.

### Identification of Genes Involved in Ethylene Signal Transduction

**FIGURE 6:**
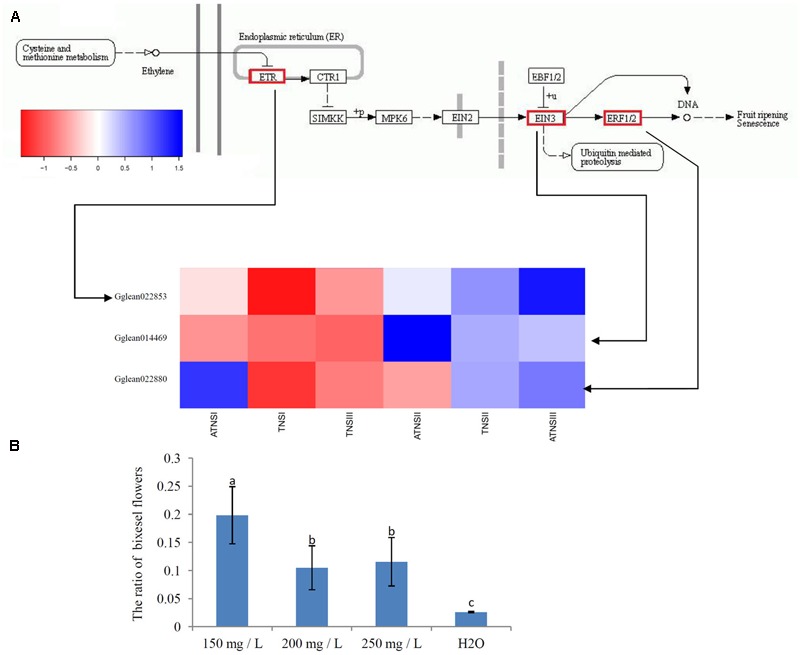
Expression of candidate genes involved in ovule development. **(A)** Expression of candidate genes involved in ethylene signal transduction. Gene expression was measured by log2Ratio. ATNSI, ATNSII, ATNSIII represented pistil of FMF when their BVD was 3.0–5.0, 5.1–13.0, and 13.1–25.0 mm. TNSI, TNSII, TNSIII represented pistil of BF when their BVD was 3.0–5.0, 5.1–13.0, and 13.1–25.0 mm. Different colors from blue to red showed the size of correlation coefficient. **(B)** Effects of different concentrations of ethylene on the ratio of BF. mg/L stands for the concentrations of ethylene. Control sprayed by water. Different small letters indicate significant difference between treatments at *P < 0.05* by Duncan’s new multiple range test. Error bar represented standard deviation.

We identified three DEGs involved in the ethylene signal transduction process: *ETR* (*Gglean022853*), *EIN3* (*Gglean14469*), and *ERF1/2* (*Gglean022880*) (**Figure 6A** and **Supplementary Table S8**). To verify the role of ethylene in FMF, we sprayed ethephon at the stage of TNSI (ATNSI) with different concentration. We found that at all concentrations used ethephon had a significant effect on the proportion of BF. With water sprayed as a control, the proportion of BF was 2.2 ± 0.00%. At 150 mg/L of ethephon, the proportion of BF was increased to 19.83 ± 0.51%, whereas at 200 and 250 mg/L ethephon the proportion was 11.5 ± 0.43 and 10.5 ± 0.39%, respectively. The results indicated that 150 mg/L was the optimal concentration for improving the proportion of BF in pomegranate (**Supplementary Table S8**). The findings using ethephon were consistent with the transcriptomics results indicating that DEGs related to ethylene response may be associated with pistil abortion in FMF (**Figure 6B**). Ethylene may act as an upstream factor for pomegranate FMF female sterility.

### qRT-PCR Analysis of Selected DEGs

**FIGURE 7:**
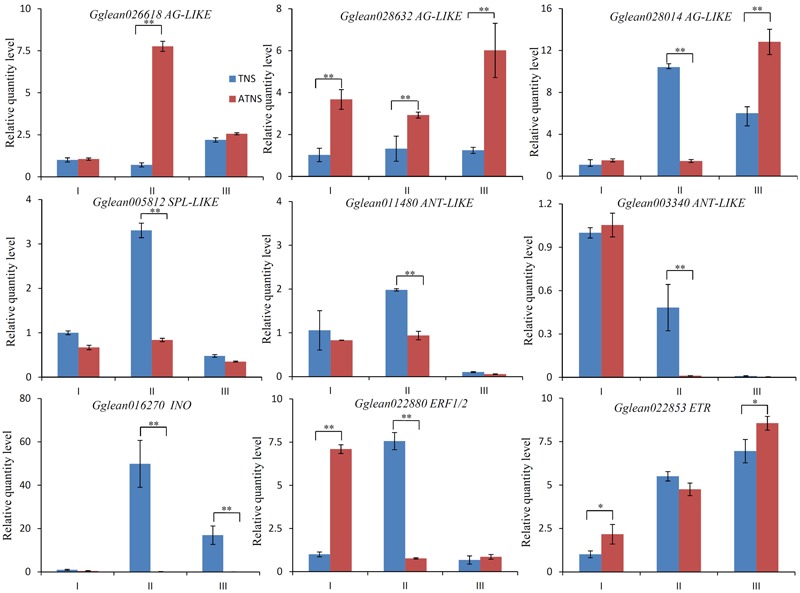
Relative quantity level of 9 selected genes at different stages of floral buds development in BF and FMF. Stage I, II, and III represents pistils of flower buds when their BVD were 3.0–5.0, 5.1–13.0, and 13.1–25.0 mm, blue column represents BF, red column represents FMF. Relative quantity levels were calculated by 2^-ΔΔC_T_^ method with actin as a standard. ^∗^Represents the level of significance difference *P* < 0.05, ^∗∗^ represents the level of significance difference *P* < 0.01 in independent-samples *t*-test.

To confirm the results of RNA sequencing, seven DEGs related to ovule development including three *AGAMOUS*-like DEGs (*Gglean026618, Gglean028632*, and *Gglean028014*), one *SPL* homolog gene (*Gglean005812*), two *ANT* homolog genes (*Gglean003340* and *Gglean011480*) and one *INO* homolog gene (*Gglean016270*) were selected for qRT-PCR analysis. In addition, two DEGs associated with ethylene signal transduction, *ETR* (*Gglean022853*) and *ERF1/2* (*Gglean022880*), were also analyzed between BF and FMF at three stages during flower development (**Figure 7**). The results showed a similar expression trend to the transcriptome analysis. Three *AGAMOUS-like* homologou*s* genes showed differing expression patterns. *Gglean026618* was up-regulated in ATNSII in the ATNSII-TNSII comparison, and *Gglean028632* displayed up-regulated in FMF in the comparisons ATNSI-TNSI, ATNSII-TNSII, and ATNSIII-TNSIII. In contrast, *Gglean028014* was down-regulated in ATNSII relative to TNSII. Similarly, the *SPL* homolog gene *Gglean005812* was down-regulated in ATNSII relative to TNSII. Two *ANT* homolog genes (*Gglean003340* and *Gglean011480*) was down-regulated in ATNSII relative to TNSII and almost no expression in TNSIII and ATNSIII. The *INO* homolog gene (*Gglean016270*) displayed almost the same trend as the *ANT* homologs. *ETR* (*Gglean022853*), the repressor of ethylene signal transduction, was up-regulated in ATNSI relative to TNSI and ATNSIII relative to TNSIII. *ERF1/2* (*Gglean022880*), the downstream gene in ethylene signal transduction pathway, displayed differential expression in both the ATNSI-TNSI and ATNSII-TNSII comparisons, being up-regulated in ATNSI relative to TNSI but down-regulated in ATNSII relative to TNSII.

## Discussion

### Nutrient Metabolism May Effect Pomegranate Female Sterility

Here, we set out to explore candidate genes associated with pistil abortion in pomegranate FMF. The key stage for pistil abortion in FMF was found to be at a BVD of 5.0–13.0 mm, when the development of the ovule ceased following the formation of the inner integument primordium. Pistil abortion is widespread in a series of fruit crops, including Japanese apricot, *X. sorbifolia*, and *Prunus armeniaca* L. (Lillecrapp et al., 1999; Gao et al., 2002; Shi et al., 2011). Although the morphological characteristics of pistil abortion are distinct between species, all instances of pistil abortion result from the abnormal development of ovules. Pomegranate FMF display termination of ovule development after the formation of the inner integument primordium, and this may be the main reason for pistil abortion in this species. In addition, in Japanese apricot it has been reported that pistil abortion may be associated with the catabolism of macromolecular nutrients in the flower buds (Shi et al., 2011). Based on module-trait co-expression analysis (**Figure 4B**), some processes related to macromolecular nutrients, including “organic substance biosynthetic process” (GO:0044711), “small molecule metabolic process” (GO:0044281), and “organ nitrogen compound metabolic process” (GO:1901564), were found to be overrepresented, suggesting that the abortion of pomegranate FMF may be related to the catabolism of nutrients (**Figure 4B**).

### Key Regulatory Factors of Ovule Development May Be Associated with Pomegranate Female Sterility

A comparative transcriptomic analysis between BF and FMF in different developmental stages was then undertaken to mine candidate genes. Significant progress in understanding the molecular mechanism of ovule development has been achieved in some species, such as *Arabidopsis*, cotton, and rice (Baker et al., 1997; Lee et al., 2006; Kubo et al., 2013). *AG, SPL, ANT, BEL1* and *INO* have been found to be key regulators of ovule development (Ito et al., 2004; Sieber et al., 2004; Ó’Maoiléidigh et al., 2013; Wei et al., 2015). In the present study, seven DEGs related to ovule development, expressed differentially at the key stage of FMF pistil abortion, have been verified by means of qRT-PCR, including three *AG* homolog genes, one *SPL* homolog, two *ANT* homologs and one *INO* homolog. Previous studies have indicated that *ANT* and *INO* are key regulators of the formation of the ovule integument (Grossniklaus and Schneitz, 1998), and here both showed down-regulation in ATNSII relative to TNSII. Furthermore, the morphology of the ovules of pomegranate FMF was similar to that of *Arabidopsis ant-72F5* ovules (Grossniklaus and Schneitz, 1998). Collectively, our results indicated that *ANT* may be a key regulator gene of pistil abortion in pomegranate FMF. *SPL* can promote the growth of ovule integuments (Balasubramanian and Schneitz, 2002). *SPL* was down-regulation in the key stage of pistil abortion (ATNSII-TNSII) in FMF, which indicated the role of *SPL* promoting pomegranate ovule development. Previous studies indicated *SPL* represses the expression of *ANT* and *INO* to control ovule development (Wei et al., 2015). However, in the present study, *SPL, ANT* and *INO* were down-regulated in ATNSII relative to TNSII, the possible explanation is that *ANT* and *INO* may be repressed by other upstream factors for pomegranate ovule abortion.

As important regulatory genes in flower development, *AG* homologs play important roles in the formation of the pistil and stamen (Bowman et al., 1991). In this study, we detected three *AG* homolog genes amongst the DEGs, of which two were up-regulated (*Gglean026618* and *Gglean028632*) and one was down-regulated (*Gglean028014*) in ATNSII relative to TNSII. The two up-regulated genes in ATNSII were *AGL19* homology gene (*Gglean026618*) and *AGL8* homology gene (*Gglean028632*). Both of these two genes play important roles in floral transition (Alvarez-Buylla et al., 2000). *AGL8* promotes reproductive transition through interaction with *SVP* and *SOC1* factors (Balanzà et al., 2014). Similarly, *AGL19* acts as a floral activator in FLC-independent vernalization pathway (Schönrock et al., 2006). Here we found the differential expression of these two genes between BF and FMF, suggesting *AGL19* and *AGL8* also play important roles in regulation of female sterility in pomegranate. The down-regulated *AG* gene was the homolog gene of *AGL62*, which can stimulate nucellus degeneration (Bertoni, 2016). Moreover, *AGL62* homology gene *Gglean028014* was up-regulated in ATNSIII relative to TNSIII. In this study, we observed ovule degradation in ATNSIII (**Figure 1**), suggesting that the up-regulation of *AGL62* may underlie the female sterility by contributing to the degradation of ovule.

### Ethylene May Act as an Upstream Factor for Pomegranate Female Sterility

Ethylene has been verified as an upstream factor for ovule development in tobacco (De Martinis and Mariani, 1999). In this study, ethylene response signal factor including *ETR* and *ERF 1/2* displayed differential expression between BF and FMF. ETR protein acts as a repressor for the transmission of ethylene signal (Hua and Meyerowitz, 1998). *ETR* (*Gglean022853*) was up-regulated in ATNSI relative to TNSI indicated the suppression of ethylene signal transduction may be a factor influencing pomegranate ovule development. Down-regulation of *ERF1/2* (*Gglean022880*) in ATNSII relative to TNSII also supported this point. Moreover, the increase of the ratio of BF in pomegranate with external ethephon treatment further confirmed the function of ethylene in pomegranate. Jointly, our findings indicated that ethylene may act as one of the upstream regulators influencing the development of pomegranate ovule. Furthermore, *ANT* contains two AP2 domains homologous with the DNA binding domain of EREBPs (Shigyo et al., 2006), The down-regulation of *ANT* may be related to ethylene.

Collectively, we indicated that *ANT* homolog gene (*Gglean003340, Gglean011480*) and *INO* homolog gene (*Gglean016270*) may act as the downstream genes that lead to ovule abortion for pomegranate FMF. *AG*-Like and *SPL* homology genes may be upstream genes impact *ANT* and *INO* expression, but there may also exist other upstream genes repressing the expression of *ANT* and *INO* in pomegranate ovule abortion. Moreover, ethylene and nutrient may be the upstream regulators that regulated some genes related to ovule development, and the optimal spraying concentration of ethephon was 150 mg / L, which can be referenced for improving the yield of pomegranate.

## Author Contributions

SC and LC conceived the project and its components. JN, HX, and LC contributed to the acquisition of materials. BL and LC accomplished the observation of embryo. LC, FZ, DZ, and QW performed spraying of ethephon and counted up the ratio of bisexual flowers. JZ, XL, and LC conducted gene expression analysis. LC and HL performed qRT-PCR, LC wrote the paper. JZ and HL helped the article proofread.

## Conflict of Interest Statement

The authors declare that the research was conducted in the absence of any commercial or financial relationships that could be construed as a potential conflict of interest.

## Footnotes

1 http://www.1gene.com.cn/

2 http://www.bioconductor.org/packages/release/bioc/html/DESeq.html

3 http://www.genome.jp/kegg/

4 http://www.cytoscape.org/

5 https://www.ncbi.nlm.nih.gov/sra?linkname=bioproject_sra_all&from_uid=381837

## Supplementary Material

TABLE S1 Statistics of pomegranate morphological index.

TABLE S2 Statistics of RNA-seq, normalized total gene read counts and expressed genes number and lists.

TABLE S3 Number of differentially expressed gene (DEG) obtained, lists of DEG, Gene Ontology enrichment of each comparison.

TABLE S4 Kyoto Encyclopedia of Genes and Genomes (KEGG) pathway of the comparison of three stages between ATNS and TNS.

TABLE S5 Modules of gene co-expression analysis.

TABLE S6 Gene Ontology enrichment of each gene module.

TABLE S7 Differentially expressed genes related to flower development which enriched by gene co-expression analysis.

TABLE S8 Differentially expressed genes related to ovule development and ethylene signal transduction.

TABLE S9 Primers of qRT-PCR.

## Abbreviations

ATNS: pistil of FMF
BF: bisexual flowers
BVD: bud vertical diameter
DEGs: differentially expressed genes
FMF: functional male flowers
GO: gene ontology
KEGG: Kyoto Encyclopedia of Genes and Genomes
OTD: ovary transverse diameter
OVD: ovary vertical diameter
SEM: scanning electron microscopy
SL: style length
TNS: pistil of BF
