# Supplementary material for: Transcriptomic Analysis Reveals Candidate Genes for Female Sterility in Pomegranate Flowers
Source: Front Plant Sci. 2017 Aug 23;8:1430. doi: 10.3389/fpls.2017.01430 (PMC5572335; doi:10.3389/fpls.2017.01430)
Supplement: Supplementary file 10 [file Image_1.PDF]

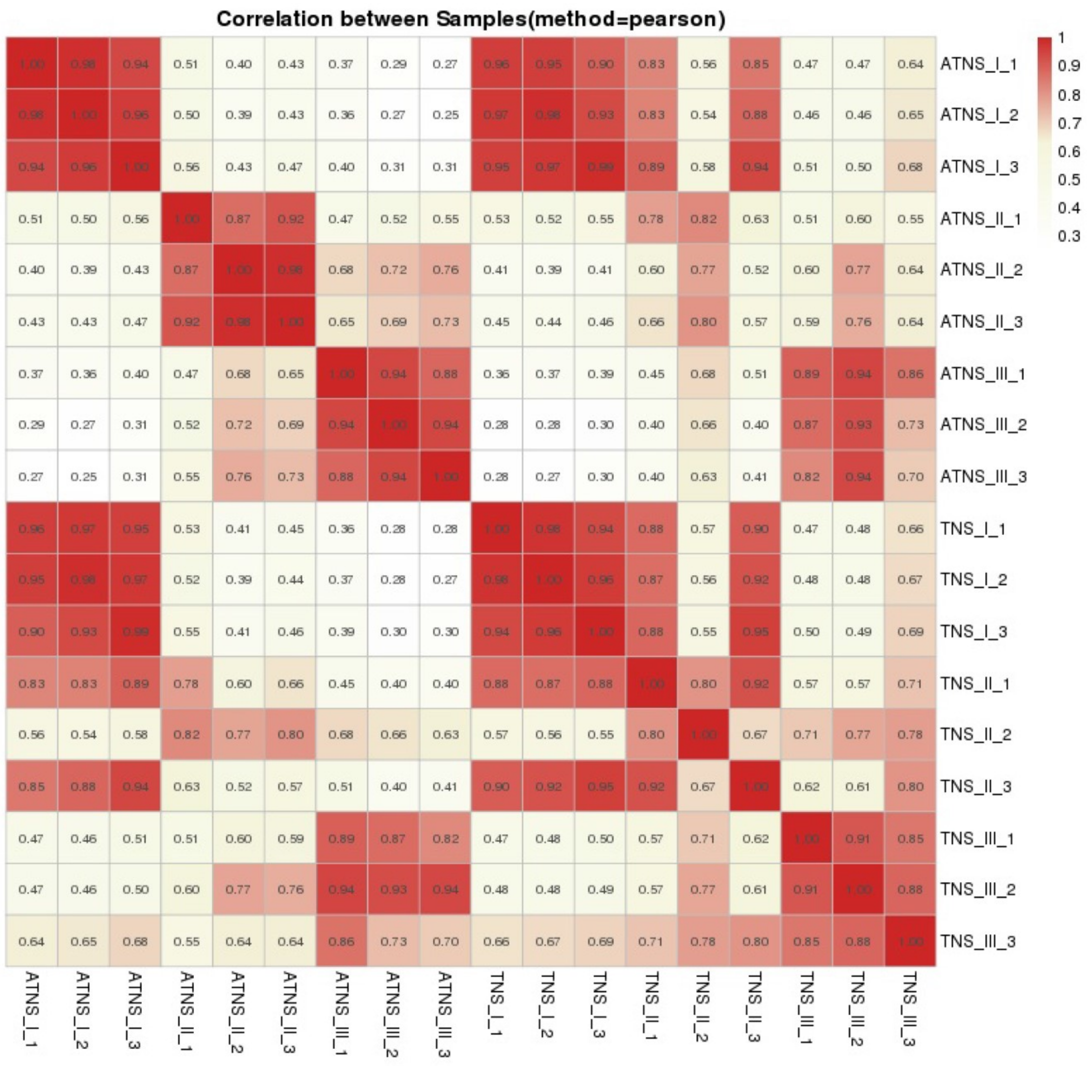

Supplementary Figure S1 Heat map of Pearson r^2 correlation values for all replicates. Each stage has three biological replicates. ATNS/TNS-I-1, ATNS/TNS-I-2, ATNS/TNS-I-3 represent three biological replicates of FMF/BF pistil in BVD 3.0-5.0 mm, respectively; ATNS/TNS-II-1, ATNS/TNS-II-2, ATNS/TNS-II-3 represent three biological replicates of FMF/BF pistil in BVD 5.1-13.0 mm, respectively; ATNS/TNS-III-1, ATNS/TNS-III-2, ATNS/TNS-III-3 stand for three biological replicates of FMF/BF pistil in BVD 13.1-25.0 mm, respectively. ATNS represents functional male flowers, TNS stands for bisexual flowers. The grids with different colors from white to red show the size of correlation coefficient, with the correlation coefficient 0-0.3, 0.3-0.4, 0.4-0.5, 0.5-0.6, 0.6-0.7, 0.7-0.8, 0.8-0.9, 0.9-1.0 represented by colors 0 to 1.

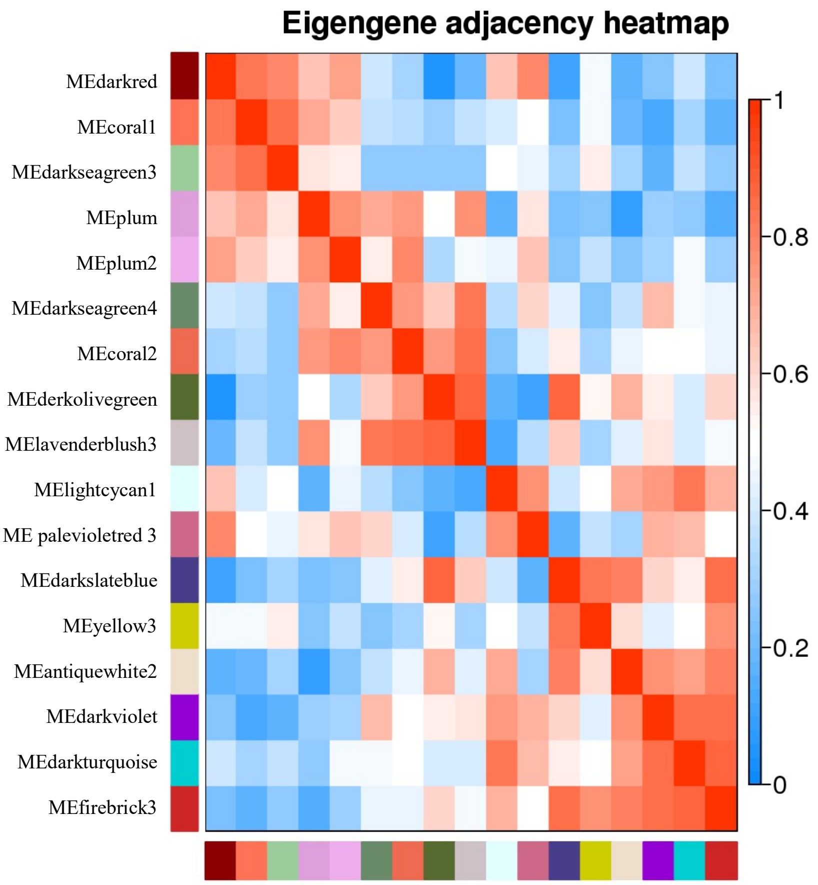

Supplementary Figure S2 Heat map of Pearson r^2 correlation values between different modules. Each row corresponds to a module. Different colors from blue to red show the size of correlation coefficient, with the correlation values 0-0.2, 0.2-0.4, 0.4-0.6, 0.6-0.8, 0.8-1.0 represented by colors 0 to 1.

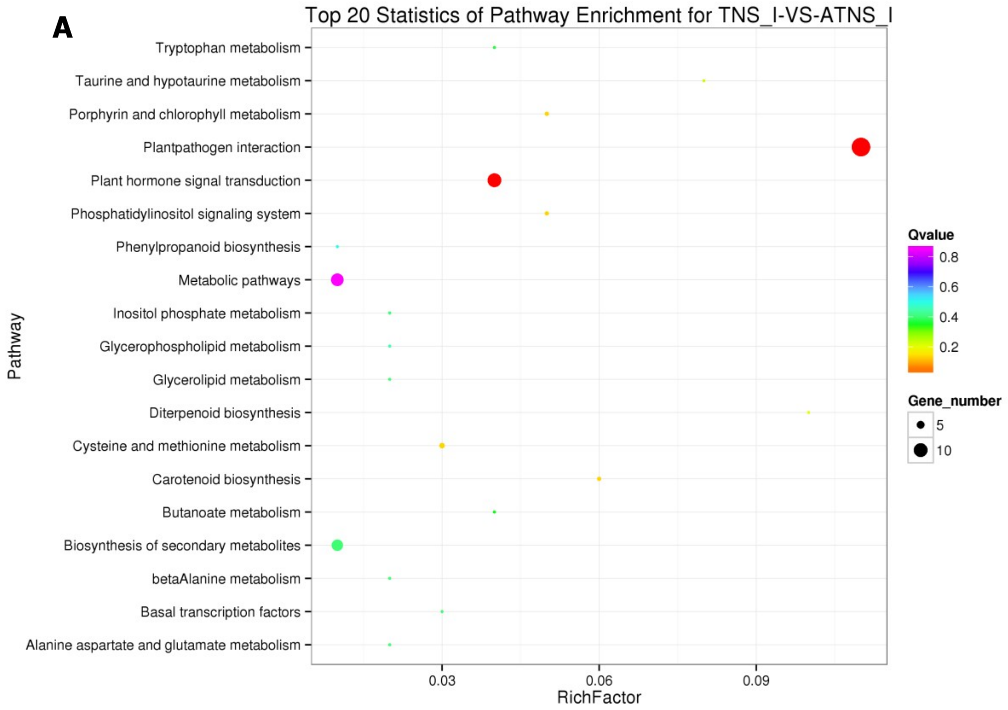

B

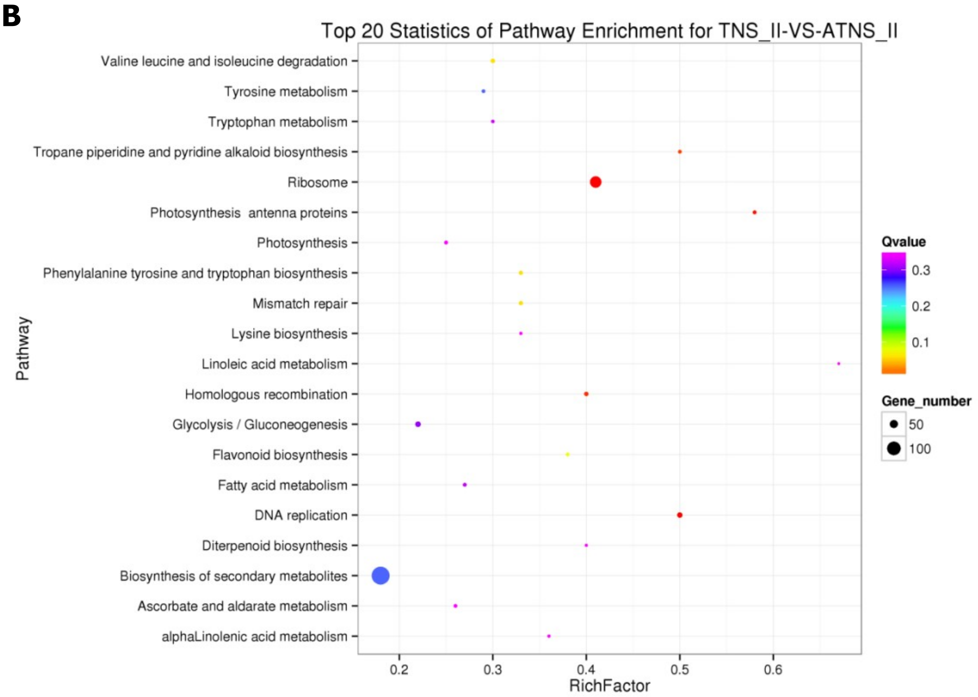

C

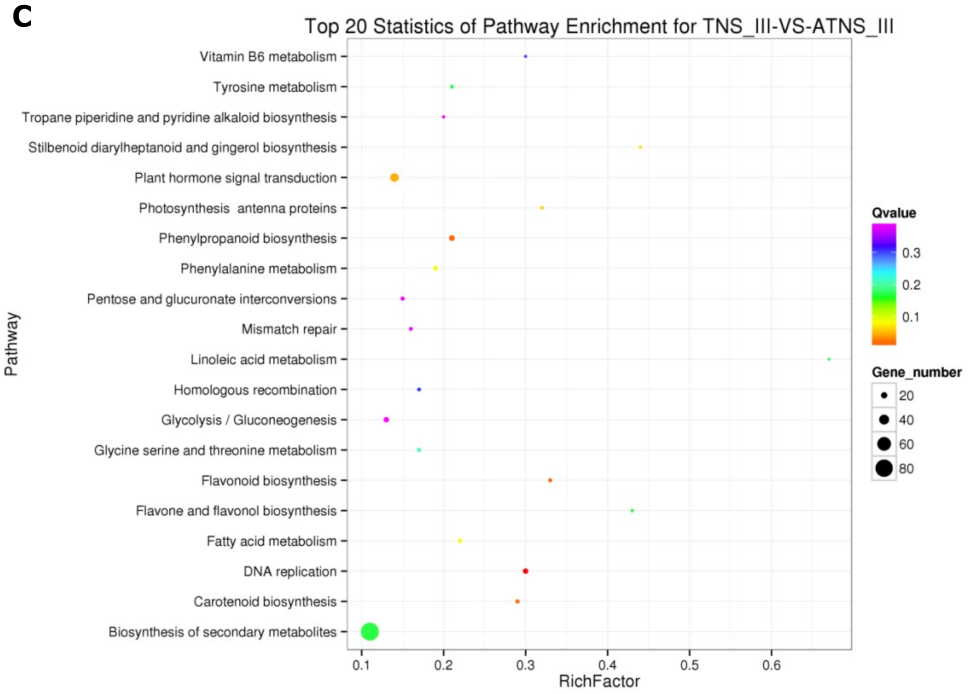

Supplementary Figure S3 Statistics of KEGG pathway enrichment. **(A)** KEGG pathway enrichment between TNAI and ATNSI; **(B)** KEGG pathway enriched between TNSII and ATNSII; **(C)** KEGG pathway enriched between TNSIII and ATNSIII.

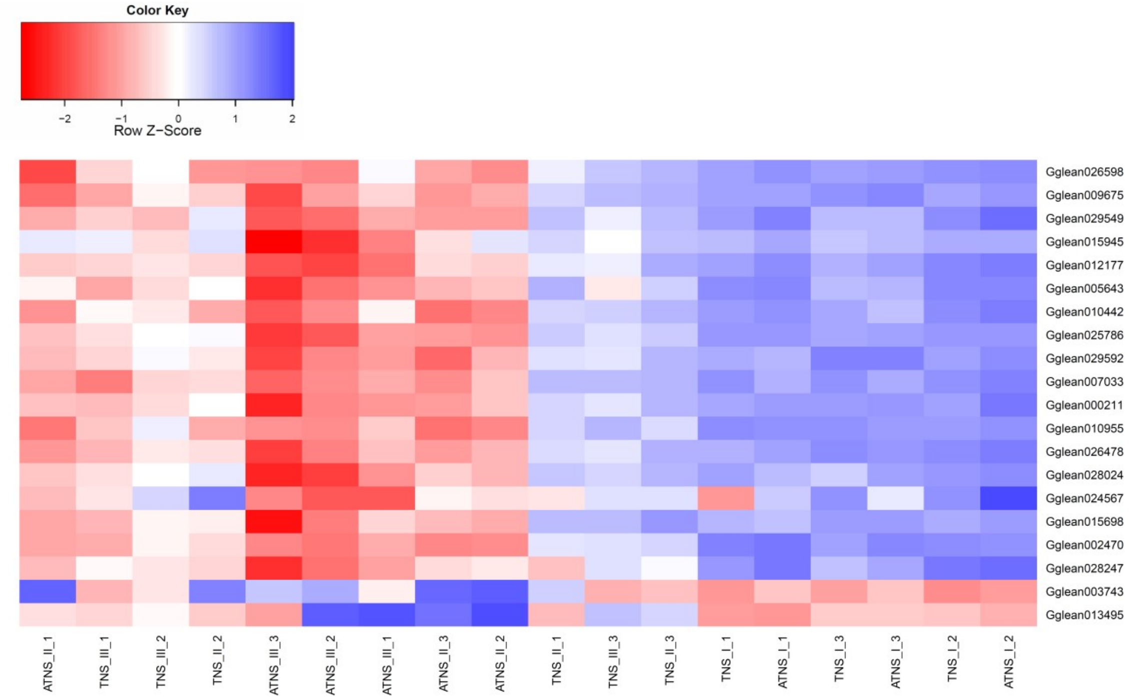

Supplementary Figure S4 Expression of candidate genes involved in flower development by co-expression analysis. Different colors from blue to red showed the relative log2 (expression ratio). ATNSI, ATNSII, ATNSIII represent pistil of FMF when their BVD was 3.0-5.0 mm, 5.1-13.0 mm and 13.1-25.0 mm. TNSI, TNSII, TNSIII represent pistil of BF when their BVD was 3.0-5.0 mm, 5.1-13.0 mm and 13.1-25.0 mm.
